# Outside the limit: questioning the distance restrictions for cooperative miRNA binding sites

**DOI:** 10.1186/s11658-023-00421-4

**Published:** 2023-01-24

**Authors:** Caroline Diener, Martin Hart, Claudia Fecher-Trost, Jessica Knittel, Stefanie Rheinheimer, Markus R. Meyer, Jens Mayer, Veit Flockerzi, Andreas Keller, Eckart Meese

**Affiliations:** 1 Institute of Human Genetics, Saarland University, 66421 Homburg, Germany; 2 Department of Experimental and Clinical Pharmacology & Toxicology, Institute of Experimental and Clinical Pharmacology and Toxicology, Center for Molecular Signaling (PZMS), Saarland University, 66421 Homburg, Germany; 3 Department of Experimental and Clinical Toxicology & Pharmacology, Institute of Experimental and Clinical Pharmacology and Toxicology, Center for Molecular Signaling (PZMS), Saarland University, 66421 Homburg, Germany; 4 Chair for Clinical Bioinformatics, Saarland Informatics Campus, Saarland University, 66123 Saarbrücken, Germany; 5 Helmholtz-Institute for Pharmaceutical Research Saarland (HIPS), Helmholtz-Centre for Infection Research (HZI), 66123 Saarbrücken, Germany

**Keywords:** microRNA, Cooperativity, Binding sites, Distances, In silico prediction, RISC, Functional interactions, Networks, Proteomics, Target identification

## Abstract

Among the concepts in biology that are widely taken granted is a potentiated cooperative effect of multiple miRNAs on the same target. This strong hypothesis contrasts insufficient experimental evidence. The quantity as well as the quality of required side constraints of cooperative binding remain largely hidden. For miR-21-5p and miR-155-5p, two commonly investigated regulators across diseases, we selected 15 joint target genes. These were chosen to represent various neighboring 3′UTR binding site constellations, partially exceeding the distance rules that have been established for over a decade. We identified different cooperative scenarios with the binding of one miRNA enhancing the binding effects of the other miRNA and vice versa. Using both, reporter assays and whole proteome analyses, we observed these cooperative miRNA effects for genes that bear 3′UTR binding sites at distances greater than the previously defined limits. Astonishingly, the experiments provide even stronger evidence for cooperative miRNA effects than originally postulated. In the light of these findings the definition of targetomes specified for single miRNAs need to be refined by a concept that acknowledges the cooperative effects of miRNAs.

**Supplementary Information:**

The online version contains supplementary material available at 10.1186/s11658-023-00421-4.

## Background

MicroRNAs (miRNAs, miRs) essentially contribute to the regulation of gene expression at the post-transcriptional level by binding via their seed region to reverse-complementary binding sites, usually located in the 3′ untranslated regions (3′UTRs) of their target mRNAs [1]. As part of the RNA-induced silencing complex (RISC), they convey an inhibition of translation or the degradation of the mRNA, ultimately resulting in reduced protein levels of their targets [2, 3]. Deregulation of miRNA expression frequently causes pathogenic alteration of cellular signaling pathways and contributes to various disease phenotypes. This renders miRNAs and their targetomes of special interest for novel diagnostic and therapeutic approaches [4–6]. The network of interactions between miRNAs and their targets is rather complex in that, a single miRNA can regulate a larger number of different targets and one target is usually regulated by several miRNAs [7].

Further complicating, it has been recognized that miRNAs may act in a cooperative manner on a shared target [7, 8]. For over almost a decade it has been established that clear distance rules apply to the cooperative function of miRNA binding sites. Since binding of multiple RISCs in proximity likely results in mutual steric hindrance, cooperative function of miRNAs is assumed to require a minimum distance of 8–13 nt between binding sites [9–11]. The maximum distance for cooperatively acting binding sites is assumed to be approximately 35–39 nt, to warrant the interaction between neighboring RISCs [9, 10]. However, those distance rules are largely based on studies that vary the distance between neighboring miRNA binding sites, for which cooperative effects have been observed. Monitoring of cooperative effects are usually done by reporter assays [9, 10]. The distance variations are mostly generated by spacer sequences of variable length that are designed to have a low tendency to form secondary and tertiary structures [9, 10]. It is, however, conceivable that secondary and tertiary structures contribute to an approaching of binding sites that are distant to each other within the linear 3′UTRs sequences of the targets [12].

Here, we set out to analyze cooperativity of miRNA binding sites in endogenous target 3′UTRs employing a high-throughput miRNA interaction reporter (HiTmIR) assay [13]. We examined 15 potential target genes with binding sites for miRNAs miR-21-5p and miR-155-5p with distances reaching from overlapping sites to neighboring sites that were up to 1300 nt distant from each other. We determined various cooperative regulatory effects for different binding sites, partially exceeding the distance rules that have former been established.

## Methods

### Prediction of miRNA binding sites

Respective 3′UTR binding sites for miR-21-5p and miR-155-5p were predicted using the miRWalk 2.0 in silico tool [14]. A site matching to at least 6 nt of the respective miRNA’s seed region was assumed for this prediction. Results were filtered for the forecast by at least four of the 13 integrated databases. The overlap of genes between the prediction for miR-21-5p and miR-155-5p was determined. For further selection of the genes to be tested by luciferase reporter assays, miRnome and transcriptome data, which have been deposited from a former project [15], were integrated as described in the results section. Expression data were evaluated as the median result from three examinations for two donors.

### Assembly of 3′-UTR reporter constructs

3′-UTR sequences of predicted miRNA targets (Additional file 2: Table S1 and Additional file 3: Table S2) were cloned into pMIR-RNL-TK dual luciferase reporter plasmid [16]. Reverse transcribed complementary DNA (cDNA) from Jurkat T-cell line (ACC282; Leibniz Institute DSMZ-German collection of microorganisms and cell cultures) was used as template. The pMIR-*DDX17*-3′UTR construct was carried on from a former project [15]. A cooperative effect of miR-21-5p and miR-155-5p on the *LEMD3*-3′UTR construct was previously shown, due to manual measurement of dual luciferase assays as part of a doctoral thesis [17].

Mutagenesis of miRNA binding sites was conducted by overlap extension PCRs [18] or alternatively by site directed mutagenesis, following the manufacturers' recommendations for Q5 Site-Directed Mutagenesis Kit (New England BioLabs Inc.; Massachusetts; United States). For the mutation of *LEMD3* 3′UTR, miRNA binding sites were replaced in equal counts of nucleotides by sites of the restriction enzymes NruI (miR-155-5p) and PmlI (miR-21-5p). For the mutation of *RECK* 3′UTR, miRNA binding sites were replaced by sites of the restriction enzymes *Nru*I (miR-155-5p BS1) and *Pml*I (miR-155-5p BS1) and *Afe*I (miR-21-5p) (Additional file 4: Table S3).

### Analysis of miRNA binding by dual luciferase reporter assays

3′UTR reporter constructs were tested as former described by liquid handling system of high-throughput miRNA interaction reporter (HiTmIR) assay [13, 15]. In variation to previous studies, HEK293T cells (Human embryonic kidney cell line; ACC635; Leibniz Institute DSMZ-German collection of microorganisms and cell cultures) were transfected with (i) 200 ng of empty pSG5 (Agilent Technologies Inc., CA, USA; for measurement of basal reporter activity), (ii) 200 ng of empty pSG5 in a 1:1 mixture with pSG5-miR-21 expression plasmid (for measurement of individual miR-21-5p effect), (iii) 200 ng of empty pSG5 in a 1:1 mixture with pSG5-miR-155 expression plasmid (for measurement of individual miR-155-5p effect) or (iv) 200 ng of a 1:1 mixture of pSG5-miR-21 and pSG5-miR-155 expression plasmids (for measurement of cooperative effects). The miRNA expression plasmids were kindly provided by Grässer et al. and former checked for their effectiveness in miRNA overexpression [17, 19]. A control of the empty pMIR reporter plasmid was included in all series of measurements. Likewise, testing sensor constructs were included as positive controls for the effectiveness of miRNA overexpression in all experiments (Additional file 5: Table S4; Additional file 1: Fig. S1).

Firefly luciferase activity was normalized in a first step to pMIR-RNL-TK encoded constitutively expressed renilla luciferase and in a second step by basal reporter activity (reporter construct and empty pSG5 co-transfection). Results were related and statistically evaluated in comparison to the empty reporter control, without the impact of any 3′UTR, for each condition of miRNA overexpression. Cooperative miRNA binding due to co-expression of miR-21 and miR-155 plasmids was evaluated in comparison to the effects of individual miRNA overexpression and classified due to a significant change to both, miR-21 and miR-155 individual overexpression. Unpaired t testing (two-side) was conducted, assuming a normal distribution of the data. All p-values were adjusted for multiple comparisons [20].

### Analysis of endogenous protein extracts by high-resolution mass spectrometry-based proteomics

HEK293T cells (2.4 × 10^6^) were seeded out in 10 cm dishes. After overnight incubation, cells were transfected with 8 µg of either (i) empty pSG5 (negative control), (ii) empty pSG5 in a 1:1 mixture with pSG5-miR-21 expression plasmid (for measurement of individual miR-21-5p effect), (iii) empty pSG5 in a 1:1 mixture with pSG5-miR-155 expression plasmid (for measurement of individual miR-155-5p effect) or (iv) a 1:1 mixture of pSG5-miR-21 and pSG5-miR-155 expression plasmids (for measurement of cooperative effects), complying with PolyFect® Transfection Reagent Handbook (09/2000; Protocol for transient transfection of 293 cells) (Qiagen, Hilden, Germany). The transfected cells were harvested after 48 h of incubation (37 °C, 5% CO_2_), resuspended in 2× denaturing cell lysis buffer (130 mM Tris/HCl, 6% SDS, 10% 3-Mercapto-1,2-propanediol, 10% glycerol) and lysed by ultrasound sonification. Whole-cell protein extracts (30 μg) were separated on NuPAGE® 4–12% gradient gels (ThermoFisher Scientific, Karlsruhe, Germany) until the bromophenol dye front reached the center of the gel. The experiment was performed with three biological replicates each. Proteins were fixed in the presence of 40% ethanol and 10% acetic acid and visualized with colloidal Coomassie stain (20% (v/v) methanol, 10% (v/v) phosphoric acid, 10% (w/v) ammonium sulfate, and 0.12% (w/v) Coomassie G-250). Six gel pieces were cut per cell lysate, washed, reduced, carbamidomethylated, and trypsin digested as described [21]. After extraction, 6 µl of tryptic peptides were analyzed by data-dependent nano liquid chromatography high-resolution mass spectrometry (nano-LC-ESI-HRMS/MS) analysis using the instrumentation: Ultimate 3000 RSLC nano system equipped with an Ultimate3000 RS autosampler coupled to with a Thermo Easy-nanoLC coupled to a Thermo Scientific Orbitrap Eclipse Tribrid mass spectrometer (Thermo Scientific, Germany). Peptides were separated by a gradient, generated with buffer A (water and 0.1% formic acid) and buffer B (90% acetonitrile and 0.1% formic acid) at a flow rate of 300 nl/min: 0–5 min 4% B, 5–80 min to 31% B, 80–95 min to 50% B, 95–100 min to 90% B, 100–105 min hold 90% B, 105–106 min to 4% B and 106–120 min to 4% B.

Peptides were trapped on a C18 trap column (75 µm × 2 cm, Acclaim PepMap100C18, 3 µm,) and separated on a reverse phase column (nano viper Acclaim PepMap capillary column, C18; 2 µm; 75 µm × 50 cm,). The chromatography effluent was sprayed into the mass spectrometer using a coated emitter (PicoTipEmitter, 30 µm, New Objective, Woburn, MA, USA, ionization energy: 2.4 keV). MS1 peptide spectra were acquired using the Orbitrap analyzer (R = 120k, RF lens = 30% m/z = 375–1500, MaxIT: auto, profile data, intensity threshold of 104). Dynamic exclusion of the 10 most abundant peptides was performed for 60 s. MS2 spectra were collected in the linear ion trap (isolation mode: quadrupole, isolation window: 1.2, activation: HCD, HCD collision energy: 30%, scan rate: fast, data type: centroid).

### Raw data analysis of mass spectrometry results

Peptides and fragments were analyzed using the MASCOT algorithm and TF Proteome Discoverer (PD) 1.4 software (ThermoFisher, Waltham, USA). Therefore, peptides were matched to tandem mass spectra by Mascot version 2.4.0 by searching of a SwissProt database (version2021_05, number of protein sequences for all taxonomies: 564.638, for taxonomy human: 20.397). Peptides were analyzed with the following mass tolerances: peptide tolerance: 10 ppm, fragment tolerance: 0.7 D. The PD workflow included tryptic digest and we allowed for up to two missed cleavage sites. Cysteine carbamidomethylation was set as a fixed modification and deamidation of asparagine and glutamine, acetylation of lysine, and oxidation of methionine were set as variable modifications. The MASCOT output files (.dat) were loaded in the software Scaffold (5, Proteome SoftwareInc., Portland, OR, USA) and separate gel bands belonging to one sample were combined with multidimensional protein-identification technology (MudPIT). To ensure significant protein identification, the false discovery rate (FDR) filter was set to 5% and 1% for protein and peptide probability, respectively. The identification of two unique peptides per protein was set as the minimum for protein identification.

### Identification of cooperative miRNA effects based on cellular proteomics data

To identify potential cooperative effects, we computed three effect sizes (Cohen’s d) for each protein. The effect size between control experiments and miR-21 overexpression (d_mir21_), between controls and miR-155 overexpression (d_mir155_) and between controls and both miRNAs overexpressed (d_mir21/155_). A potential cooperative effect was defined as max(d_mir21_, d_mir155_) + 2 < (d_mir21/155_) and (d_mir21/155_) > 1. The latter criterion ensures that we observe an actual reduction in the respective protein by miRNA overexpression, while the first criterion ensures that the effect in overexpressing both miRNAs is substantially exceeding the maximal reduction effect for the overexpression of the two miRNAs separately.

To match the seed sequences to the 3′ UTRs, we used the Bioconductor GenomicFeatures package. We downloaded the transcripts and extracted the 3′ UTRs using the makeTxDbFromUCSC and the three UTRsByTranscript function. Then we searched for all exact matches of all seed sequences and binding patterns in the 3′ UTR. This analysis was done for the two miRNAs independently. Next, we computed for each 3′ UTR the minimal distance between any pair of binding sites in the 3′ UTRs.

### In silico prediction of 3′UTR sequence folding

Prediction of secondary structure folding for 3′UTR sequences was conducted by using the Vienna package RNAfold tool as implemented in Geneious 2022.1 (Biomatters Ltd., Auckland, New Zealand). The 3′UTR sequences, as assayed in HiTmIR, were imported into Geneious as fasta sequences. RNA secondary structures were predicted based on energy models of "Turner 2004" [22] and using parameters as follows: Calculate a partition function and base pairing probability matrix, do not exclude GU pairs, avoid isolated base pairs, assume linear molecule, dangling ends at both sides, and temperature 37 °C. Binding sites of miR-21-5p and miR-155-5p were identified and annotated within the 3′UTR sequences.

## Results

### Selection of cooperative miRNAs and targets

Cooperativity is suggested to be a broad, but so far little explored phenomenon, which may apply to many different miRNAs [12, 23]. As for the selection of miRNAs, we chose miR-21-5p and miR-155-5p that have been reported as potent regulators for a large number of targets with special relevance in various contexts of health and disease [24, 25]. In our recent study on the early human T cell activation process, we highlighted miR-21-5p and miR-155-5p as highly expressed miRNAs with a shared target network and a likely cooperative function [15]. Furthermore, a cooperatively regulated targeting in mice argues for the evolutionary conservation of a cooperative interaction of these two miRNAs [26].

**Fig. 1:**
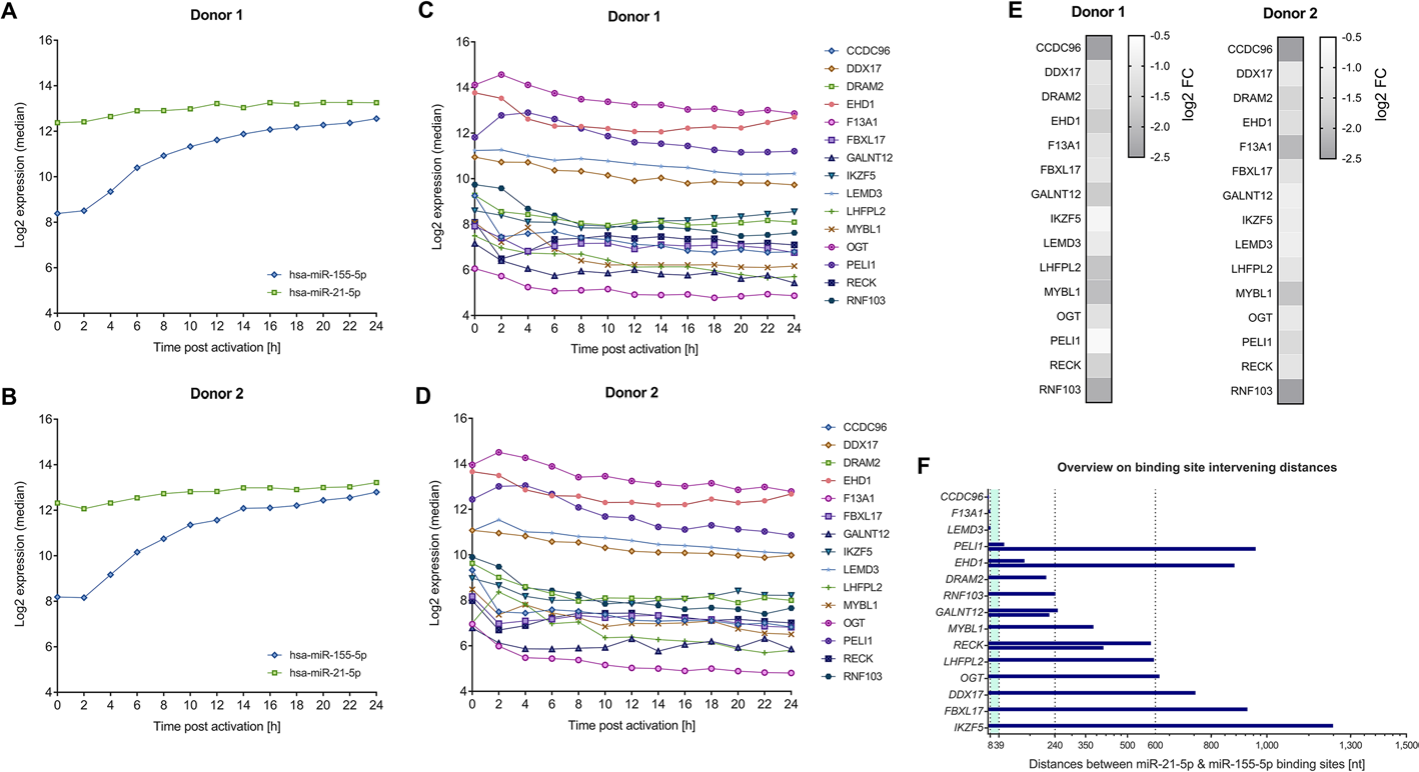
Parameters of cooperative target genes that were selected for the analysis of cooperative miRNA effects by dual luciferase reporter assays. **A**–**E** RNA expressional data from a time-course study on the early T cell activation process [15] were utilized for the selection of 15 putative joint targets of miR-21-5p and miR-155-5p to be tested by HiTmIR dual luciferase reporter assays. The hsa-miR-21-5p and hsa-miR-155-5p showed a strong expressional increase upon T cell activation (Donor 1 (**A**), Donor 2 (**B**)) and the mRNAs of the selected genes showed expressional decrease during the time-course in both analyzed donors (Donor 1 (**C**), Donor 2 (**D**)). Maximum mRNA expression changes compared to the 0 h time-point (log_2_ fold changes (FCs)) are depicted for the 15 selected genes (**E**). **F** The length of distances between in silico predicted binding sites for miR-21-5p and miR-155-5p are given for the 3’UTR sequences of the selected genes. The previously defined distance range for cooperative miRNA binding is highlighted in turquoise color

As for miRNA target sequences, we set out to identify sequences that are targets to both miR-21-5p and miR-155-5p. We used the miRWalk 2.0 tool [14] for an in silico prediction of endogenous 3′UTRs binding sites for both, miR-21-5p and miR-155-5p. We considered only putative targets that were predicted by at least four of the 13 algorithms integrated in miRWalk 2.0 and identified 1295 shared target genes for miR-21-5p and miR-155-5p. We next related the expressional data of the two miRNAs and to their potential targets utilizing information from our former study (as mentioned above) that integrated miRnome and transcriptome data in context with early T cell activation process [15]. Since both miRNAs showed strong expressional increase upon T cell activation (Fig. 1A and B), we selected only genes, the mRNAs of which showed an according expressional decrease (Fig. 1C and D). Out of 355 identified potential targets, we chose a total of 15 genes with log_2_ fold changes ranging from − 0.66 to − 2.50 after T cell activation as compared to the value prior to activation (Fig. 1E). Furthermore, the 15 targets were selected to represent various 3′UTR binding site constellations for miR-21-5p and miR-155-5p, ranging from partially (1 nt of miR-155-5p/ 3 nt of miR-21-5p) overlapping binding sites to neighboring binding sites that were up to 1,300 nt apart from each other (Fig. 1F). As summarized in Additional file 2: Table S1 the sequences were also selected to contain multiple binding sites for either of the two miRNAs.

### The effect of single and simultaneous miRNA expression

To screen for the effect of differing binding distances, we cloned corresponding 3′UTR sequences into pMIR-RNL-TK dual luciferase reporter plasmid. Resulting plasmids were employed in the semi-automated HiTmIR assay [13] to examine effects upon overexpression of only miR-21-5p or only miR-155-5p as well as co-expression of both miRNAs (Additional file 6: Table S5A), notably in the same experiment, thus allowing for better comparability between different conditions. The same amounts of transfected miRNA expression plasmids were used for comparisons between single and co-expression effects. Four independent experiments were performed in technical duplicates each. MiRNA induced effects were evaluated based on averaged values relative to an empty reporter plasmid control. All p-values were adjusted by the Benjamini–Hochberg procedure to control for false discovery rate (FDR) [20]. We categorized target sequences based on the distances between neighboring miR-21-5p and miR-155-5p binding sites within the 3′UTRs of the target genes. In detail, we differentiated between genes with miRNA binding sites that were either < 40 nt, 40–240 nt, > 240–600 nt, or > 600 nt apart from each other.

**Fig. 2:**
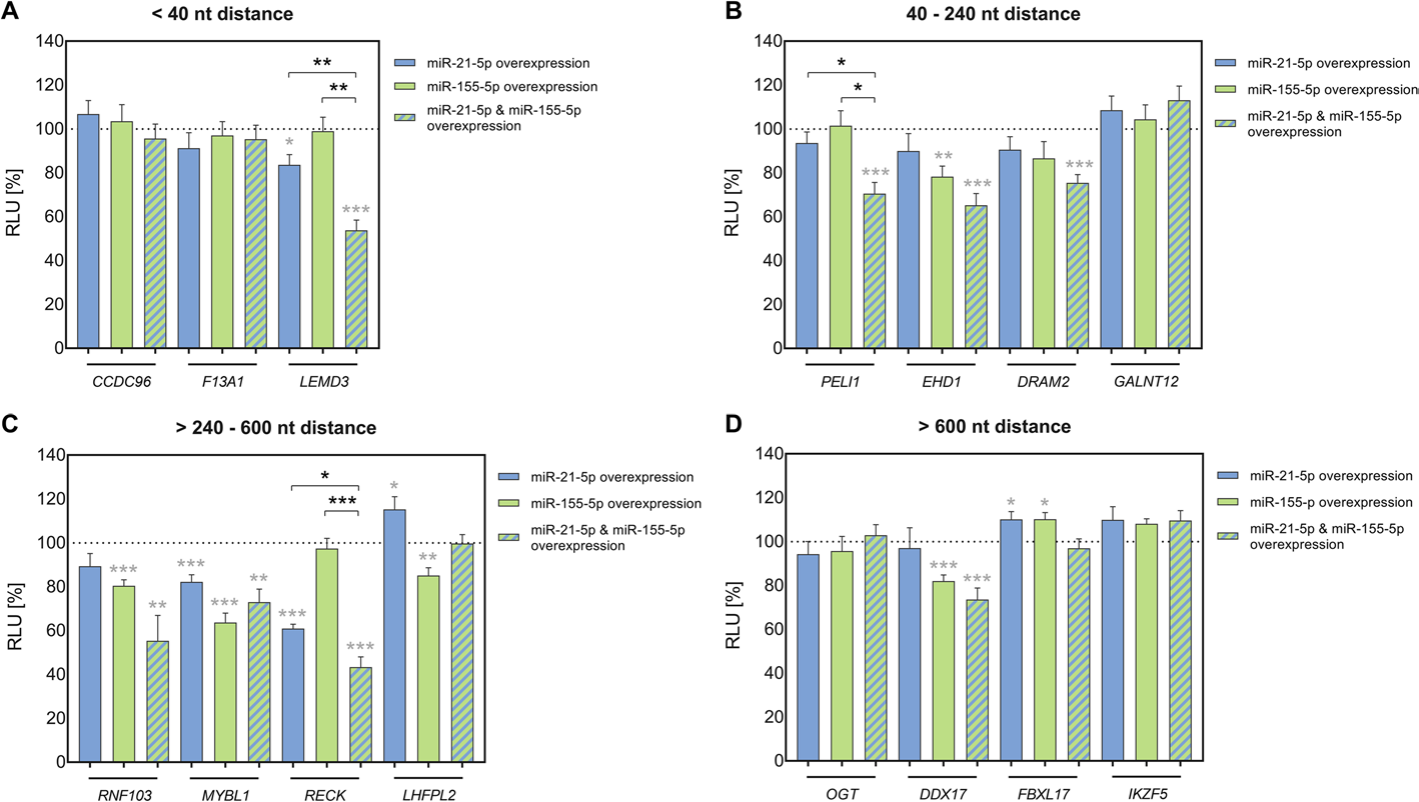
Analysis of miRNA cooperativity by HiTmIR assays. The 3’UTR sequences of 15 putative cooperative targets of miR-21-5p and miR-155-5p were cloned into pMIR-RNL-TK reporter plasmid and analyzed by semi-automated HiTmIR dual luciferase reporter assays. MiRNA induced effects on firefly luciferase activity were determined upon overexpression of only miR-21-5p or only miR-155-5p or upon co-expression of both miRNAs. For representation, the analyzed genes were categorized into four groups regarding the distances between neighboring miR-21-5p and miR-155-5p binding sites (< 40 nt (**A**), 40-240 nt (**B**), > 240–600 nt (**C**), > 600 nt (**D**)). Relative luciferase units (RLU) were related to the activity of an empty reporter control (pMIR-RNL-TK; under the respective miRNA condition) and are shown as the mean result (± standard error of the mean (± SEM)) of four independent experiments that were conducted in technical duplicates. Statistical evaluation was performed in comparison to the empty reporter control (gray asterisks) or for the co-expressional conditions in comparison to the effects upon single miRNA overexpression of miR-21-5p and miR-155-5p (black asterisks), respectively. Significant p-values after FDR adjustment are denoted (*p ≤ 0.05, **p < 0.01, *** p < 0.001)

A first category of genes (*CCDC96*, *F13A1* and *LEMD3*) (Fig. 2A) represented predicted miR-21-5p and miR-155-5p binding sites less than 40 nt apart from each other.

The 3′UTR of *CCDC96* showed partially overlapping binding sites for miR-21-5p and miR-155-5p. Neither miR-21-5p, nor miR-155-5p or co-expression of both miRNAs produced a significant regulatory effect. That result is consistent with the idea that very close proximity of miRNA binding sites causes mutual steric hindrance of RISCs [11].

The *F13A1* 3′UTR was characterized by an 8 nt distance between the two miRNA binding sites. Although that distance is assumed to be sufficient for cooperative effects, there were no regulatory effects from single expression or co-expression of both miRNAs.

In contrast, the *LEMD3* 3′UTR, with the two miRNA binding sites laying in 9 nt distance from each other, showed no effect for miR-155-5p, but an effect for miR-21-5p and an even more pronounced effect for co-expression of both miRNAs. Specifically, relative luciferase reporter activity, measured as relative light units (RLU), was not significantly affected by miR-155-5p expression compared to empty reporter control (RLU = 98.98 ± 6.35; p = 0.998). Overexpression of miR-21-5p, however, significantly reduced the RLU to 83.64% ± 4.64 (p = 2.27 × 10^–2^) and co-expression of both miRNAs further reduced the RLU to 53.76% ± 4.66 (p = 6.34 × 10^–6^). The effect of the co-expression of both miRNAs was also significantly different from the effects measured separately for each of the miRNAs (miR-21-5p: p = 4.61 × 10^–3^; miR-155-5p: p = 1.02 × 10^–3^).

A second category of genes, including *PELI1, EHD1, DRAM2,* and *GALNT12* (Fig. 2B), displayed distances between neighboring miR-21-5p and miR-155-5p binding sites ranging from 40 nt to 240 nt.

The *PELI1* 3′UTR showed no effects for neither miR-21-5p nor miR-155-5p (miR-21-5p: RLU = 93.62 ± 5.00; p = 0.439; miR-155-5p: RLU = 101.46 ± 6.84; p = 0.894), but significant decrease of RLU to 70.51% ± 5.14 (p = 7.59 × 10^–4^) was detectable, when co-expressing both miRNAs. The effect of the co-expression of both miRNAs was also significantly different from the effects measured for each of the miRNAs (miR-21-5p: p = 2.45 × 10^–2^; miRNA-155: p = 1.86 × 10^–2^). In contrast to our results, separate regulatory effects have formerly been described of each the miR-21-5p and the miR-155-5p on *PELI1* in mice [27, 28]. These contrary findings may be explained by experimental differences regarding the size of the analyzed 3′UTR sequences, by the different species and by a different miRNA dose [27, 29].

The *EHD1* 3′UTR showed no effect for miR-21-5p, but a significant effect for miR-155-5p, and an even more pronounced effect when co-expressing both miRNAs. However, this effect that could indicate some cooperative interaction, was not significantly verified when compared to the single miRNA´s effects on *EDH1* 3′UTR. Likewise, *DRAM2* 3′UTR showed significant effect on RLU upon co-expression of both miRNAs, but no significant difference to the effect of the single miRNAs. As for the *GALNT12* 3′UTR, neither expression of single nor co-expression of both miRNAs resulted in a significant regulatory effect.

A third category of genes, including *RNF103, MYBL1, RECK,* and *LHFPL2* (Fig. 2C), displayed distances between neighboring miR-21-5p and miR-155-5p binding sites ranging from 240 to 600 nt.

As for the *RNF103* gene, miR-21-5p on its own did not have an effect on the RLU, but co-expression with miR-155-5p contributed to increase the effects of the miR-155-5p. As for the *EDH1* 3′UTR and the *DRAM2* 3′UTR potential cooperative effects could not be statistically verified within the scope of our measurements.

Similar to above described *LEMD3, RECK* showed no effect for miR-155-5p (RLU = 97.40 ± 4.68; p = 0.692), but an effect for miR-21-5p (RLU = 60.94% ± 2.00; p = 7.46 × 10^–10^), and an even more pronounced effect, when co-expressing both miRNAs (RLU = 43.33% ± 4.68; p = 6.64 × 10^–8^). The effect of the co-expression was also significantly different from the effects measured for each of the miRNAs (miR-21-5p: p = 3.81 × 10^–2^; miR-155-5p: p = 2.14 × 10^–5^). The other genes in this category, specifically *MYBL1* and *LHFPL2*, did not show a cooperative effect upon combined overexpression of miR-21-5p and miR-155-5p.

The fourth category of genes, including *OGT*, *DDX17*, *FBXL17*, and *IKZF5*, displayed distances greater than 600 nt between miR-21-5p and miR-155-5p binding sites (Fig. 2D).

Only the *DDX17* 3′UTR showed a relatively mild regulatory effect for miR-155-5p and a likewise mild cooperative non-significant effect for the co-expression of both miRNAs.

The other genes in this category, specifically *OGT*, *FBXL17*, and *IKZF5*, showed no regulatory effect from single or co-expression of miRNAs.

Several of the above described genes were newly identified as targets for individual miR-155-5p and miR-21-5p. *EHD1* and *RNF103* were identified as new targets of miR-155-5p and *MYBL1* as a target of both miR-155-5p and miR-21-5p, respectively. Likewise, previously reported effects were consistent with our data, including the formerly described regulation of *RECK* by miR-21-5p [30] and the regulations of *LHFPL* and *DDX17* by miR-155-5p [15, 31]. The increases of RLU that were detected for some genes, including *LHFPL* upon miR-21-5p overexpression and *FBXL17* upon individual overexpression of both miRNAs, may be attributable to some secondary effects, potentially from the regulation of endogenous targets affecting the reporter construct activity.

### Validation of cooperative effects by reporter assays and potential impact of RNA secondary structures

We chose two of the identified cooperative targets for exemplary validation of the identified binding effects. We also evaluated the possible impact of secondary structures on these binding effects. Thereto, we predicted secondary structures for corresponding 3′UTR sequences using RNAfold as implemented in Geneious v2022.1 [22]. In detail, we analyzed *LEMD3* and *RECK* 3′UTRs that both displayed cooperative effects for miR-21-5p and miR-155-5p within the initial luciferase assays. While *LEMD3* sequence included neighboring binding sites of miR155-5p and miR-21-5p (5′ → 3′) at a distance of 9 nt, corresponding to the former defined limits, *RECK* 3′UTR included neighboring binding sites at a distance of 414 nt. Besides, *RECK* 3′UTR included an additional miR-155-5p binding site (BS1) at a distance of 584 nt in 5′ direction to miR-21-5p binding site.

**Fig. 3:**
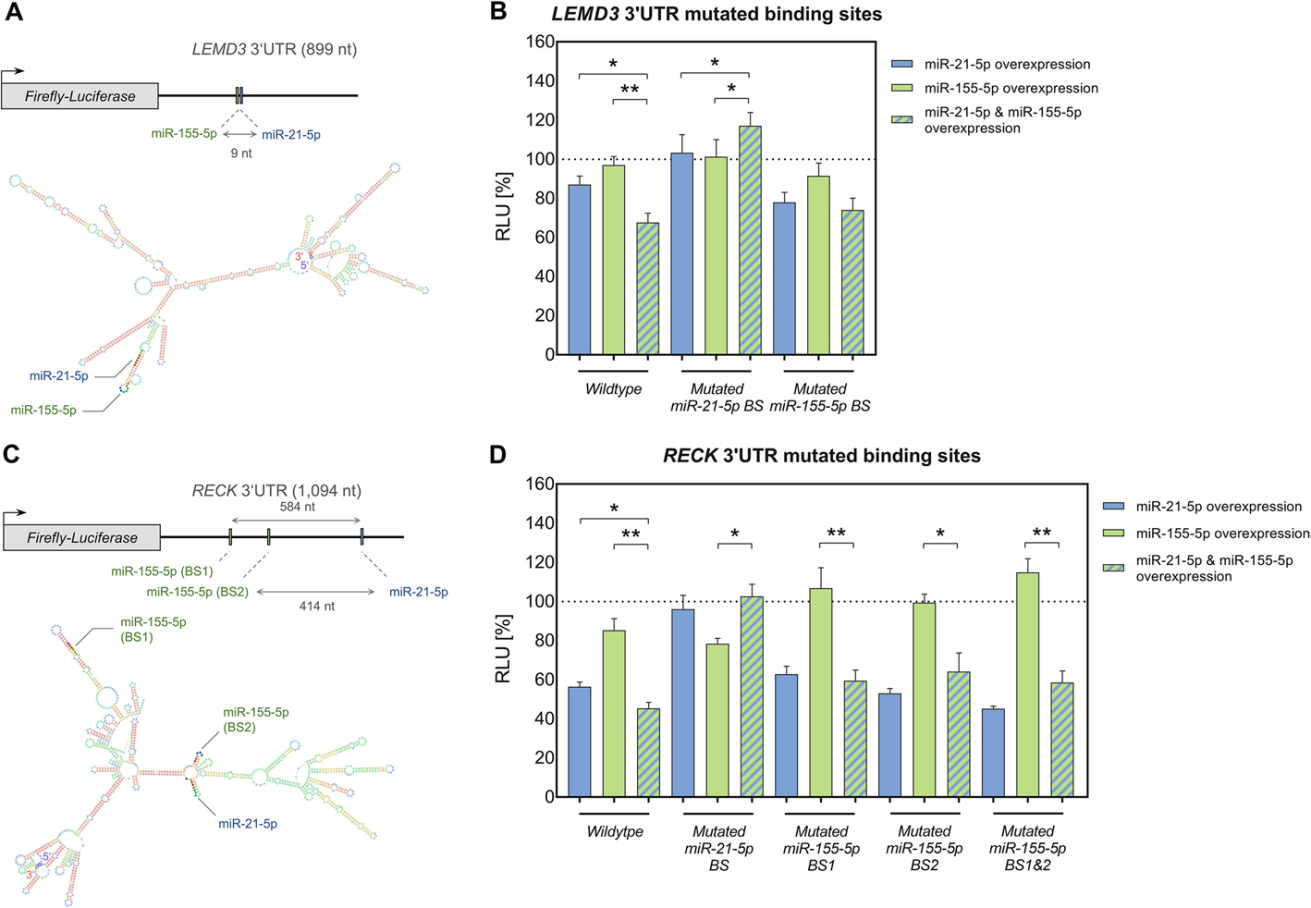
In silico prediction of 3’UTR secondary structures and exemplary validation miRNA cooperative effects. **A**, **C**: RNA secondary structures were predicted for *LEMD3* (**A**) and *RECK* (**C**) 3′UTR sequences that showed cooperative binding of miR-21-5p and miR-155-5p as determined by the HiTmIR dual luciferase assays. Corresponding 3′UTR sequences (as cloned for the underlying assays) are shown in a linearized (upper panel; 5′→3′) and in a folded representation (lower panel), respectively. The included miRNA binding sites are indicated. **B**, **D** For the validation of miRNA cooperative effects, miR-21-5p and miR-155-5p binding sites were mutated within pMIR-*LEMD3* (**B**) and pMIR-*RECK* (**D**) 3′UTRs. The reporter constructs were tested by HiTmIR dual luciferase reporter assays. Comparative measurements were conducted with both, the wild type and mutated reporter constructs. MiRNA induced effects were determined upon overexpression of only miR-21-5p or only miR-155-5p or upon co-expression of both miRNAs. Relative luciferase units (RLU) were related to the activity of an empty reporter control (pMIR-RNL-TK; under the respective miRNA condition) and are shown as the mean result (± standard error of the mean (± SEM)) of three independent experiments that were conducted in technical duplicates. Statistical evaluation of the cooperative effects was performed in comparison to the effects upon single miRNA overexpression of miR-21-5p or miR-155-5p (black asterisks). Significant p-values after FDR adjustment are denoted (*p ≤ 0.05, **p < 0.01, ***p < 0.001)

Predicting RNA secondary structures for *LEMD3* indicates that both miRNA's binding sites locate within the same hairpin structure. Hence, the sequence proximity and the orientation could allow for an interaction between two binding RISCs (Fig. 3A).

To validate the previously detected effects on the wildtype sequence, miRNA binding sites within the *LEMD3* 3′UTR reporter constructs were mutated. As compared to the single miRNA´s effects, the reporter assays demonstrated reversal of the cooperative miRNA effect upon mutation of either the miR-21-5p or the miR-155-5p binding site (Fig. 3B; Additional file 6: Table S5B). These findings highlight the relevance of both binding sites for the induction of the miRNA cooperative effect. Notably, in the case of miR-21-5p mutation, even an opposite development towards a slight increase of RLU could be detected as compared to the single miRNA´s effects.

As for the structure prediction on *RECK* 3′UTR sequence (Fig. 3C), the binding sites of miR-21-5p and miR-155-5p at 414 nt distance (BS2) may be in a spatial proximity due to their localization on two small opposite hairpin structures that are connected by a small common circular base. Under the assumption of certain 3-dimensional motility of these arms, a cooperative interaction is conceivable. According to our prediction of RNA secondary structures, the additional upstream miR-155-5p binding site (BS1), which is located at an even greater nucleotide distance to miR-21-5p, lies at the upper end of a larger secondary structure. A potential 3-dimensional movement of this protruding structure may permit a binding site approximation leading to a cooperative interaction.

Reporter assays verified the cooperative miRNA binding on *RECK* wild type 3′UTR, as defined by a significant decrease of RLU in comparison to the two miRNA´s individual effects, and showed reversal of the miRNA cooperative effect upon mutation of the included miR-21-5p and miR-155-5p binding sites (Fig. 3D; Additional file 6: Table S5B). According to our reporter assay results, no clear statement can be made about the relevance of the two included miR-155-5p binding sites. A reversion of cooperativity was achieved upon all three conditions, mutation of miR-155-5p BS1, miR-155-5p BS2 and the mutation of both miR-155-5p binding sites (BS1&BS2). In principle, a kind of ping-pong mechanism would be imaginable, in which both of the miR-155-5p binding sites could provide alternating interaction with the miR-21-5p binding site.

### Cooperative miRNA effects on protein targets with distant 3′UTR binding sites

We examined cooperative miRNA effects on endogenous protein levels using the abovementioned pSG5-based miRNA expression system in 293T human embryonic kidney cells as cellular model for mass spectrometry-based protein analyses. As for the luciferase assays, we analyzed the cellular protein extracts following co-expression of both miRNAs or following the overexpression of either miR-21-5p or miR-155-5p. Three independent experiments were performed and a transfection with the empty effector plasmid was carried out as control. As detailed in the methods section, cooperative effects were determined by the comparison of effect sizes.

**Fig. 4:**
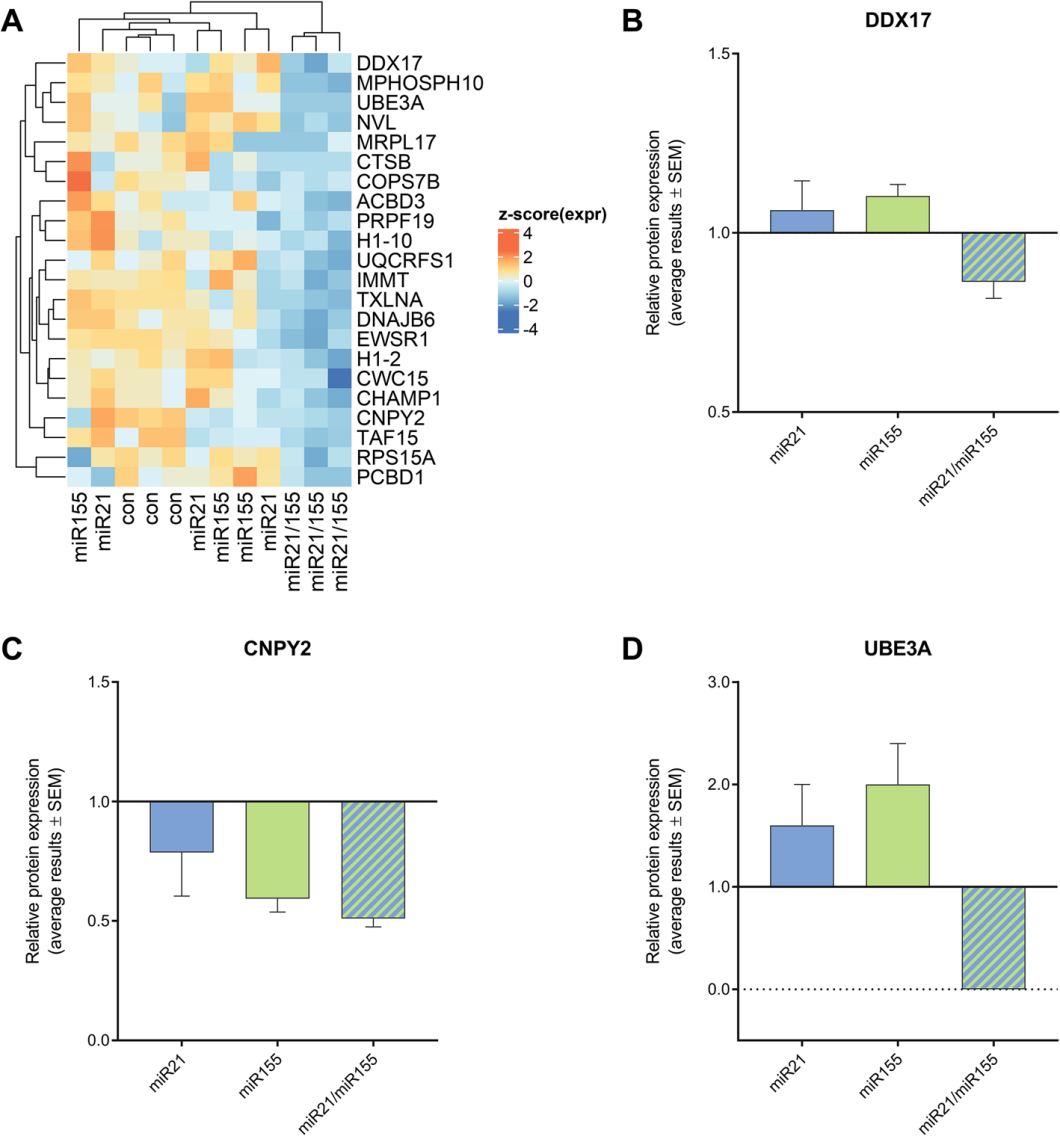
Analysis of miRNA cooperativity by proteome data. Whole cell protein extracts from 293T human embryonic kidney cells were analyzed by high-resolution mass spectrometry upon (i) control plasmid transfection (con), (ii) overexpression of only miR-21-5p (miR21), (iii) only miR-155-5p (miR155) or (iv) upon co-expression of both miRNAs (miR21/155). Three independent transfection experiments were performed. As defined by the comparison of effect sizes by Cohen’s d, 22 gene products indicated cooperative miRNA effects (**A**; clustering of z-scored expression data). For specific representation, relative protein expression data for DDX17 (**B**), CYPN2 (**C**) and UBE3A (**D**) were normalized to controls and are shown as averaged results with SEM

Three genes *(DDX17, EHD1* and *LEMD3),* for which cooperative effects or trends have been observed by the luciferase assays, were identified in the proteome analyses i.e., were among the detected proteins in HEK293T cells (Additional file 7: Table S6). As defined by the comparison of Cohen’s d (see methods), *DDX17* showed a cooperative effect of miR-21-5p and miR-155-5p by the proteomics analysis (Fig. 4A and B). The proteome data also indicated cooperative effects for 21 additional proteins (Fig. 4A), including CNPY2 and UBE3A (Fig. 4C and D) that contained seed binding sites for both miRNAs within their 3′UTR transcript sequences. In detail, the 3′UTR sequence of CNPY2 contained three miR-21-5p binding-sites and one miR-155-5p site at a minimal distance of at least 158 nt. The 3′UTR sequence of UBE3A contained only one binding site for each miRNA at a distance of 208 nt. In both cases, the distances of the binding sites exceeded the previously defined limits of 39 nt.

## Discussion

Analyzing endogenous target sequences by high-throughput luciferase assays, we identified significant cooperative effects of miR-155-5p and miR-21-5p for three out of 15 tested genes (*LEMD3*, *PELI1* and *RECK*). While the binding sites for *LEMD3* mapped within formerly defined distance limits, the binding sites within the 3′UTRs of both *PELI1* and *RECK* were separated by much greater distances.

The approach of luciferase reporter assay preselects those 3′UTRs that show binding sites for the two miRNAs. Our proteome analyses in contrast do not involve any a priori selection. Additionally, different cell types served as starting points for our reporter based binding tests (T-cell genes) and for our analyses on endogenous protein levels (embryonic kidney cells). As independent confirmation, our proteomics data support the idea of cooperative miRNA binding beyond the previously defined distance limits for neighboring 3′UTR binding sites.

Overall, our observations raise the general question, whether strict distance rules can be applied or whether additional parameters determine the effects of cooperative binding sites. Such additional factors may need to be considered for an effective screening of cooperative miRNA effects. As for the nature of such factors, our structural predictions together with the mutagenesis tests provide a first hint on the likely impact of mRNA secondary structure. Future in-depth analyses by structural biology will finally be needed to specify the determinants that allow for cooperative miRNA binding.

There is evidence that the genes that are cooperatively regulated by miR-155-5p and miR-21-5p play an integral role in T-cell activation networks. *LEMD3* gene product for example, is a known antagonist of the TGF-β signaling [32, 33], which is important for the coordination of a wide variety of T cell functions [34, 35]. Notably, LEMD3 protein was also represented by few spectral counts in our proteomic data of embryonic kidney cells. Although, cooperativity of miR-21-5p and miR-155-5p co-expression has not ultimately be proven in this case, these protein data provide further evidence for such an effect. Extended proteomic analyses will help to clarify to what extend cooperative effects consistently occurs in different cell types.

Besides *LEMD3*, *PELI1* encodes a ubiquitin ligase that is considered as a negative regulator of T cell activity [36]. Likewise, the RECK protein negatively regulates various matrix metalloproteinases, the expression of which is of major importance for an effective T cell response [37–39]. In addition to these target genes, there are likely other genes that are also regulated in a coordinated manner by the co-expression of two and more miRNAs as part of the T-cell activation networks.

In case of the *LEMD3* and *RECK* 3′UTR sequences, binding of miR-155-5p did not have an influence on its own but contributed to increase the effects miR-21-5p. This observation is consistent with a scenario in which the binding of a specific miRNA-coupled RISC promotes recruitment of an additional RISC, coupled to another miRNA [8]. Moreover, our results on *PELI1* show that miRNA cooperativity can occur without a measurable effect for either single miRNA. The requirement of two miRNAs to achieve a functional mRNA binding complicates the definition of miRNA targets, which is as of now based on the idea that a specific miRNA regulates a respective target. This might be a rather simplified view, and further studies must consider that the effects of a specific miRNA on a target should only be defined if the effects of other miRNAs that bind to the same mRNA target are also acknowledged. Our findings have also a bearing on the definition of tragetomes.

Besides the cooperative effects of miR-155-5p and miR-21-5p on *LEMD3*, *PELI1* and *RECK* we also found evidence for cooperative miRNA interaction on four further genes including *EHD1*, *DRAM2*, *RNF103* and *DDX17*. These findings were, however, not statistically verified by our measurements. One has, nevertheless, to be careful not to discount these findings. The assumed cooperative effect on e.g., *DDX17* was supported by our analyses at the embryonic kidney cell protein level. Functionally DDX17 represents an RNA helicase that is involved in the regulation of multiple transcription factors [40, 41] and in the processing of other miRNAs [42]. The cooperative regulation of such a central gene emphasizes the important impact of cooperative miRNA effects on the regulation of cellular networks. In addition, the identified cooperative effects on proteins that lack common binding sites for miR-21-5p and miR-155-5p could indicate a regulatory layer of indirect cooperative effects, which may be confirmed by future functional network analyses.

## Conclusion

In summary, our data show cooperative scenarios for miRNAs that bind within the same endogenous 3′UTR of a target gene. Binding of one miRNA can enhance the binding effects of the other miRNA, even if this miRNA does not have an influence on its own. Likewise, miRNA cooperativity can occur without an individual miRNA effect. Remarkably, miRNA cooperative effects can be found for binding sites beyond the limits of distance rules, which have been held valid for over almost a decade. Our results highlight that special attention should be paid to miRNA cooperativity and that future analyses of miRNA targetomes need to include cooperative effects of miRNAs directed to the same target. This has not only consequences for strategies to define miRNA functions as part of the basic research, but also for the employment of miRNAs clinical contexts, such as the application of miRNA-therapeutics. Our analyses provide a base for future studies on distance limitations of cooperative miRNA binding sites, the structural determinants of underlying mechanisms and the consistency of corresponding effects between different cell types.

## Supplementary Information

**Additional file 1: Figure S1.** Controls for HiTmIR based analyses of 3’UTR reporter constructs.

**Additional file 2: Table S1.** Genomic localization of 3’UTR sequences that were cloned into pMIR-RNL-TK reporter plasmid for the testing of miRNA-target-interactions by dual luciferase assays.

**Additional file 3: Table S2. **Primer sequences for the cloning of 3’UTR reporter constructs.

**Additional file 4: Table S3.** Primer sequences that were utilized for the mutation of miRNA binding sites within 3’UTR reporter constructs.

**Additional file 5: Table S4. **Sequences of sensors for miR-21-5p and miR-155-5p were utilized as positive controls on the effectiveness of miRNA overexpression in dual luciferase reporter assays.

**Additional file 6: Table S5. **Summarized results of high-throughput dual luciferase reporter assays, testing the effects of single miR-21-5p or miR-155-5p overexpression or the effect of a combined overexpression on shared predicted target genes.

**Additional file 7: Table S6.** Analysis of cooperative miRNA effects by high-resolution mass spectrometry-based proteomics.

## Data Availability

The data generated or analyzed during this study are included in this published article and its supplementary information files. Further questions can be directed to the corresponding author.

## Abbreviations

3′UTR: 3′ Untranslated region
BS number: Binding site number (5′ → 3′)
cDNA: Complementary DNA
d_miRNA_: Cohen’s d in context with miRNA overexpression
FDR: False discovery rate
HiTmIR assay: High-throughput miRNA interaction reporter assay
miRNA/miR: MicroRNA
MudPIT: Multidimensional protein-identification technology
nano-LC-ESI-HRMS/MS: Nano liquid chromatography high-resolution mass spectrometry
PD software: Proteome Discoverer software
RISC: RNA-induced silencing complex
RLU: Relative light units
