# Supplementary material for: Outside the limit: questioning the distance restrictions for cooperative miRNA binding sites
Source: Cell Mol Biol Lett. 2023 Jan 24;28:8. doi: 10.1186/s11658-023-00421-4 (PMC9875415; doi:10.1186/s11658-023-00421-4)
Supplement: Supplementary file 1 — Additional file 1: Figure S1. Controls for HiTmIR based analyses of 3’UTR reporter constructs. [file 11658_2023_421_MOESM1_ESM.pdf]

Figure S1

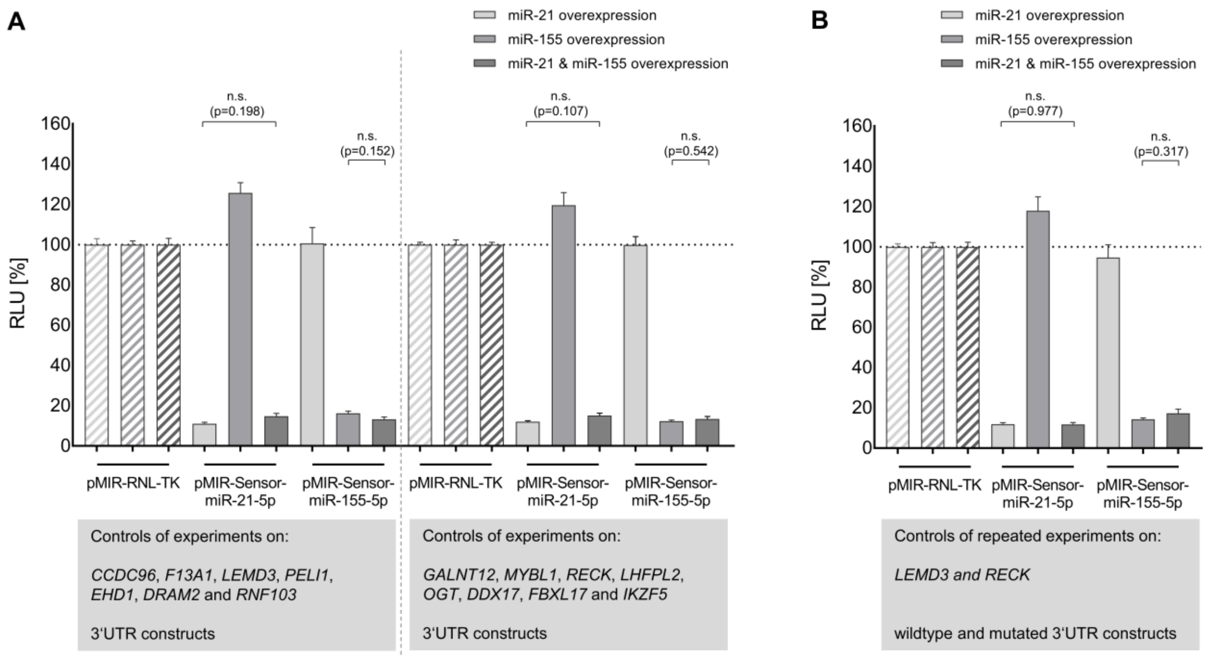

Supplementary Figure S1: Controls for HiTmIR based analyses of 3'UTR reporter constructs. For the identification of 3'UTR related miRNA effects, results of HiTmIR dual luciferase assays were set in relation to a control of empty reporter plasmid (pMIR-RNL-TK) that was carried under the conditions of individual miRNA overexpression (miR-21 or miR-155) or under co-expression of both miRNAs, respectively. Additionally, sensor constructs for miR-21-5p and miR-155-5p (as specified in Supplementary Table S4) were carried as positive controls for the effectiveness and uniformity of miRNA overexpression within all experiments of dual luciferase reporter assays. (**A**) The controls of the initial measuring series are shown as mean results of four independent experiments that were conducted in technical duplicates, (**B**) controls of the repeated measuring series (integrating the mutated 3'UTR constructs) are shown as mean results of three independent experiments that were conducted in technical duplicates, including the corresponding SEMs. Statistical results (unpaired t test, FDR adjusted) are indicated for the comparison between i) single miR-21 overexpression and its co-expression with miR-155 for pMIR-Sensor-miR-21-5p or between ii) single miR-155 overexpression and its co-expression with miR-21 for pMIR-Sensor-miR-155-5p, respectively.
