# Supplementary material for: Outside the limit: questioning the distance restrictions for cooperative miRNA binding sites
Source: Cell Mol Biol Lett. 2023 Jan 24;28:8. doi: 10.1186/s11658-023-00421-4 (PMC9875415; doi:10.1186/s11658-023-00421-4)
Supplement: Supplementary file 2 — Additional file 2: Table S1. Genomic localization of 3’UTR sequences that were cloned into pMIR-RNL-TK reporter plasmid for the testing of miRNA-target-interactions by dual luciferase assays. [file 11658_2023_421_MOESM2_ESM.pdf]

Table S1: Genomic localization of 3'UTR sequences that were cloned into pMIR-RNL-TK reporter plasmid for the testing of miRNA-target-interactions by dual luciferase assays. Information on predicted miR-155-5p and miR-21-5p binding sites is included.

Potential target gene	Genomic localization of the cloned 3'UTR sequence (GRCh38)	Included binding sites (5'→ 3')		
CCDC96	Chr4:7,040,852-7,041,276	miR-155-5p (7mer-A1)	miR-21-5p (7mer-A1)	
F13A1	Chr6:6,144,414-6,145,549	miR-155-5p (7mer-m8)	miR-21-5p (6mer)	
LEMD3	Chr12:65,246,665-65,247,508	miR-155-5p (7mer-A1)	miR-21-5p (7mer-m8)	
PELI1	Chr2: 64,093,577-64,094,708	miR-21-5p (8mer)	miR-155-5p (7mer-A1)	miR-155-5p (6mer)
EHD1	Chr11:64,852,699-64,853,760	miR-21-5p (8mer)	miR-155-5p (6mer)	miR-155-5p (7mer-A1)
DRAM2	Chr1:111,117,159-111,118,146	miR-155-5p (7mer-m8)	miR-21-5p (7mer-A1)	
GALNT12	Chr9: 98,849,153-98,849,963	miR-21-5p (7mer-m8)	miR-155-5p (7mer-A1)	miR-21-5p (6mer)
RNF103	Chr2:86,616,519-86,617,585	miR-21-5p (6mer)	miR-155-5p (7mer-m8)	
MYBL1	Chr8:66,562,209-66,563,215	miR-21-5p (7mer-A1)	miR-155-5p (8mer)	
RECK	Chr9:36,123,276-36,124,383	miR-155-5p (7mer-A1)	miR-155-5p (6mer)	miR-21-5p (8mer)
LHFPL2	Chr5: 78,485,987-78,487,074	miR-21-5p (6mer)	miR-155-5p (7mer-A1)	
OGT	ChrX: 71,574,017-71,575,205	miR-21-5p (7mer-A1)	miR-155-5p (7mer-A1)	
DDX17	Chr22:38,483,505-38,484,538	miR-21-5p (7mer-m8)	miR-155-5p (7mer-A1)	
FBXL17	Chr5:107,859,239-107,860,453	miR-21-5p (7mer-A1)	miR-155-5p (6mer)	
IKZF5	Chr10:122,990,889-122,992,237	miR-21-5p (7mer-A1)	miR-155-5p (6mer)	
