# Supplementary material for: Outside the limit: questioning the distance restrictions for cooperative miRNA binding sites
Source: Cell Mol Biol Lett. 2023 Jan 24;28:8. doi: 10.1186/s11658-023-00421-4 (PMC9875415; doi:10.1186/s11658-023-00421-4)
Supplement: Supplementary file 3 — Additional file 3: Table S2. Primer sequences for the cloning of 3’UTR reporter constructs. [file 11658_2023_421_MOESM3_ESM.pdf]

Table S2: Primer sequences for the cloning of 3’UTR reporter constructs.

The denoted primer pairs were utilized for the amplification of 3’UTR sequences of putative miRNA targets. The amplified sequences were subsequently cloned into pMIR-RNL-TK reporter plasmid for testing by dual luciferase reporter assays.

**Putative target gene**	**Forward primer sequence (5'→3')** (including *Spe*I restriction site for plasmid insertion)	**Reverse primer sequence (5'→3')** (including *Sac*I restriction site for plasmid insertion)
CCDC96	GACTAGTCAGATCGATGAGAAGAG	CGAGCTCCCAAAGAATTTAATAATTAG
DRAM2	GACTAGTGTAATGATTATGATTCTCAGGG	CGAGCTCCACACTTATTTTGCATGTAG
EHD1	GACTAGTCTCCGACGCGCAACC	CGAGCTCGAAAAGACAAGGAAGCAC
F13A1	GACTAGTGCTCTTGCTTTGACTTAGG	CGAGCTCCACACCCTAGACATATCAG
FBXL17	GACTAGTCAATTAATTCATAATAGGGAC	CGAGCTCCACATGTATTTATGACATTC
GALNT12	GACTAGTGCTAAGCAGTGACCAGAAC	CGAGCTCCACAAACTCTAACGCTG
IKZF5	GACTAGTGAAAGCCACTGTAATGAG	CGAGCTCCTGCTTTCCGGAATC
LHFPL2	GACTAGTGTATCTTCATAGCAGCCTAGG	CGAGCTCGACTCCTGTCCAGTTTCC
MYBL1	GACTAGTGACCACCACTTGCACTGTG	CGAGCTCCAACATTGTATGCAAGATTC
OGT	GACTAGTCAGATGGTGCATAGGTCTGG	CGAGCTCGCAACTGGATAGCGTAAG
PELI1	GACTAGTCAGACCATTGTCTTGCAGG	CGAGCTCCAACTGTCTATACTTGTGGTG
RECK	GACTAGTGACCACTGCCATATGATTTAC	CGAGCTCGCAATTGCAACAAGCATGC
RNF103	GACTAGTGCCTGAAACCGTGGATAG	CGAGCTCCCATAGCAGTATCAAGAAAG
