# Supplementary material for: Outside the limit: questioning the distance restrictions for cooperative miRNA binding sites
Source: Cell Mol Biol Lett. 2023 Jan 24;28:8. doi: 10.1186/s11658-023-00421-4 (PMC9875415; doi:10.1186/s11658-023-00421-4)
Supplement: Supplementary file 4 — Additional file 4: Table S3. Primer sequences that were utilized for the mutation of miRNA binding sites within 3’UTR reporter constructs. [file 11658_2023_421_MOESM4_ESM.pdf]

Table S3: Primer sequences that were utilized for the mutation of miRNA binding sites within 3'UTR reporter constructs.

The miR-21-5p and miR-155-5p binding sites within 3'UTR constructs were mutated by overlap extension PCR or site directed mutagenesis, respectively, using the denoted oligonucleotide primers. In case of overlap extension PCR, the denoted primers were combined with peripheral primer pairs that were utilized for the cloning of the wildtype construct.

**Generated 3'UTR construct**	**Template plasmid**	**Forward primer for mutagenesis (5'→3')**	**Reverse primer for mutagenesis (5'→3')**
pMIR-LEMD3_Mutated miR-21-5p_BS (a)	pMIR-LEMD3	GGATGTCTGCATTAAAGCAGTAAACACGTGGTTCCATTTTATTC	GAATAAAATGGAACCACGTGTTTACTGCTTTAATGCAGACATCC
pMIR-LEMD3_Mutated miR-155-5p_BS (a)	pMIR-LEMD3	GGATGTCTTCGCGAGAGCAGTAAAATAAGCTTTCCATTTTATTC	GAATAAAATGGAAAGCTTATTTTACTGCTCTCGCGAAGACATCC
pMIR-RECK_Mutated miR-21-5p_BS (b)	pMIR-RECK	GTTTGAACAGCGCTGTTTGATGTAATAC	TGTGAAACACATTCAAAGTATTCAATTTCTTAGAC
pMIR-RECK_Mutated miR-155-5p_BS1 (b)	pMIR-RECK	CAAATTTTCGCGAGATTACAGAAATTTAATTC	CCTGATAATTTACATAATTTACATAATTTCAAAG
pMIR-RECK_Mutated miR-155-5p_BS2 (a)	pMIR-RECK	CATCAAAGCATCTTCACGTGTGCAATTTTTATATTAAC	GTTAATATAAAAATTGCACACGTGAAGATGCTTTGATG
pMIR-RECK_Mutated miR-155-5p_BS1&2 (a)	pMIR-RECK_Mutated miR-155-5p_BS1		

(a) overlap extension PCR

(b) site directed mutagenesis
