# Supplementary material for: Outside the limit: questioning the distance restrictions for cooperative miRNA binding sites
Source: Cell Mol Biol Lett. 2023 Jan 24;28:8. doi: 10.1186/s11658-023-00421-4 (PMC9875415; doi:10.1186/s11658-023-00421-4)
Supplement: Supplementary file 5 — Additional file 5: Table S4. Sequences of sensors for miR-21-5p and miR-155-5p were utilized as positive controls on the effectiveness of miRNA overexpression in dual luciferase reporter assays. [file 11658_2023_421_MOESM5_ESM.pdf]

Table S4: Sequences of sensors for miR-21-5p and miR-155-5p were utilized as positive controls on the effectiveness of miRNA overexpression in dual luciferase reporter assays.

The denoted sensor oligonucleotides were annealed and cloned into pMIR-RNL-TK plasmid via *Spe*I and ***Sac*I** restriction sites. For effective miRNA binding, the sensor sequences included two reverse-complementary binding sequences of full-length hsa-miR-21-5p or hsa-miR-155-5p, respectively.

	**Sensor sequence (5'→3')**
miR-21-5p sensor forward strand	ACTAGTGGTGACAGGGACTATCTATGTCAACATCAGTCTGATAAGCTAGGTGACAGGGACTATCTATGTCAACATCAGTCTGATAAGCTAGGTGACAGGGACTATCTATG**GAGCTC**
miR-21-5p sensor reverse strand	**GAGCTC**CATAGATAGTCCCTGTCACCTAGCTTATCAGACTGATGTTGACATAGATAGTCCCTGTCACCTAGCTTATCAGACTGATGTTGACATAGATAGTCCCTGTCACCACTAGT
miR-155-5p sensor forward strand	ACTAGTGTGACAGGGACTATCTATGAACCCCTATCACGATTAGCATTAAGGTGACAGGGACTATCTATGAACCCCTATCACGATTAGCATTAAGTGACAGGGACTATCTATG**GAGCTC**
miR-155-5p sensor reverse strand	**GAGCTC**CATAGATAGTCCCTGTCACTTAATGCTAATCGTGATAGGGGTTCATAGATAGTCCCTGTCACCTTAATGCTAATCGTGATAGGGGTTCATAGATAGTCCCTGTCACACTAGT
