# Supplementary material for: Outside the limit: questioning the distance restrictions for cooperative miRNA binding sites
Source: Cell Mol Biol Lett. 2023 Jan 24;28:8. doi: 10.1186/s11658-023-00421-4 (PMC9875415; doi:10.1186/s11658-023-00421-4)
Supplement: Supplementary file 6 — Additional file 6: Table S5. Summarized results of high-throughput dual luciferase reporter assays, testing the effects of single miR-21-5p or miR-155-5p overexpression or the effect of a combined overexpression on shared predicted target genes. [file 11658_2023_421_MOESM6_ESM.pdf]

Table S5: Summarized results of high-throughput dual luciferase reporter assays, testing the effects of single miR-21-5p or miR-155-5p overexpression or the effect of a combined overexpression on shared predicted target genes.

A) For the initial testing, four independent experiments were conducted in technical duplicates. (RLU: Relative luciferase activity in relation to an empty control of the pMIR-RNL-TK reporter plasmid; SEM: Standard error of the mean; adj. p value: p values after adjustment by the method of Benjamini and Hochberg)

		RLU [%]	SEM	adj. p value compared to pMIR reporter control	adj. p value compared to combined overexpression
pMIR-CCDC96	miR-21-5p overexpression	106.76	6.16	0.469	0.298
	miR-155-5p overexpression	103.47	7.62	0.731	0.526
	miR-21-5p & miR-155-5p overexpression	95.58	6.66	0.657	
pMIR-F13A1	miR-21-5p overexpression	91.21	7.03	0.424	0.710
	miR-155-5p overexpression	97.05	6.30	0.731	0.849
	miR-21-5p & miR-155-5p overexpression	95.30	6.44	0.638	
pMIR-LEMD3	miR-21-5p overexpression	83.64	4.64	0.023	4.607E-03
	miR-155-5p overexpression	98.98	6.35	0.908	1.022E-03
	miR-21-5p & miR-155-5p overexpression	53.76	4.66	6.345E-06	
pMIR-PELI1	miR-21-5p overexpression	93.62	5.00	0.439	0.0245
	miR-155-5p overexpression	101.46	6.84	0.894	0.0186
	miR-21-5p & miR-155-5p overexpression	70.51	5.14	7.590E-04	
pMIR-EHD1	miR-21-5p overexpression	89.93	8.00	0.424	0.064
	miR-155-5p overexpression	78.26	4.85	2.335E-03	0.156
	miR-21-5p & miR-155-5p overexpression	65.20	5.40	2.788E-04	
pMIR-DRAM2	miR-21-5p overexpression	90.53	5.96	0.309	0.108
	miR-155-5p overexpression	86.60	7.67	0.216	0.297
	miR-21-5p & miR-155-5p overexpression	75.46	3.76	6.781E-04	
pMIR-RNF103	miR-21-5p overexpression	89.38	5.80	0.232	0.064
	miR-155-5p overexpression	80.41	2.78	1.971E-04	0.108
	miR-21-5p & miR-155-5p overexpression	55.31	11.61	5.424E-03	
pMIR-GALNT12	miR-21-5p overexpression	108.55	6.49	0.310	0.734
	miR-155-5p overexpression	104.37	6.64	0.642	0.513

		RLU [%]	SEM	adj. p value compared to pMIR reporter control	adj. p value compared to combined overexpression
	miR-21-5p & miR-155-5p overexpression	113.13	6.42	0.105	
pMIR-MYBL1	miR-21-5p overexpression	82.23	3.23	5.890E-04	0.352
	miR-155-5p overexpression	63.66	4.35	1.806E-05	0.379
	miR-21-5p & miR-155-5p overexpression	72.97	5.96	1.649E-03	
pMIR-RECK	miR-21-5p overexpression	60.94	2.00	7.464E-10	3.813E-02
	miR-155-5p overexpression	97.40	4.68	0.692	2.139E-05
	miR-21-5p & miR-155-5p overexpression	43.33	4.68	6.640E-08	
pMIR-LHFPL2	miR-21-5p overexpression	115.22	5.82	0.044	0.117
	miR-155-5p overexpression	85.10	3.57	0.011	0.107
	miR-21-5p & miR-155-5p overexpression	99.68	4.10	0.967	
pMIR-OGT	miR-21-5p overexpression	94.27	5.80	0.481	0.416
	miR-155-5p overexpression	95.67	6.69	0.642	0.525
	miR-21-5p & miR-155-5p overexpression	102.93	4.82	0.642	
pMIR-DDX17	miR-21-5p overexpression	96.98	9.31	0.801	0.117
	miR-155-5p overexpression	82.01	2.80	8.175E-04	0.352
	miR-21-5p & miR-155-5p overexpression	73.58	5.24	8.175E-04	
pMIR-FBXL17	miR-21-5p overexpression	110.11	3.61	0.038	0.115
	miR-155-5p overexpression	110.23	2.98	0.038	0.107
	miR-21-5p & miR-155-5p overexpression	96.96	4.30	0.642	
pMIR-IKZF5	miR-21-5p overexpression	109.87	6.05	0.198	0.992
	miR-155-5p overexpression	108.09	2.33	0.055	0.855
	miR-21-5p & miR-155-5p overexpression	109.61	4.53	0.102	

B) For the repeated testing of *LEMD3* and *RECK*, including binding site mutated 3'UTR constructs, three independent experiments were conducted in technical duplicates.

(RLU: Relative luciferase activity in relation to an empty control of the pMIR-RNL-TK reporter plasmid; SEM: Standard error of the mean; adj. p value: p values after adjustment by the method of Benjamini and Hochberg)

		**RLU [%]**	**SEM**	**adj. p value compared to pMIR**	**adj. p value compared to combined overexpression**
pMIR-LEMD3 (wildtype)	miR-21 overexpression	87.08	4.29	3.245E-02	2.900E-02
	miR-155 overexpression	96.98	4.37	0.692	5.783E-03
	miR-21 & miR-155 overexpression	67.66	4.76	2.968E-04	
pMIR-LEMD3_miR-21-5p_BSmut	miR-21 overexpression	103.29	9.28	0.800	3.471E-01
	miR-155 overexpression	101.24	8.79	0.896	2.720E-01
	miR-21 & miR-155 overexpression	117.05	6.87	0.065	
pMIR-LEMD3_miR-155-5p_BSmut	miR-21 overexpression	77.97	5.06	4.574E-03	0.636
	miR-155 overexpression	91.50	6.50	0.342	0.124
	miR-21 & miR-155 overexpression	74.08	6.01	5.193E-03	
pMIR-RECK (wildtype)	miR-21 overexpression	56.40	2.40	2.236E-07	3.482E-02
	miR-155 overexpression	85.31	5.87	0.065	1.003E-03
	miR-21 & miR-155 overexpression	45.42	3.02	2.995E-07	
pMIR-RECK_miR-21-5p_BSmut	miR-21 overexpression	96.07	7.03	0.716	0.567
	miR-155 overexpression	78.42	2.73	2.821E-04	1.553E-02
	miR-21 & miR-155 overexpression	102.66	6.16	0.792	
pMIR-RECK_miR-155-5p_BS1mut)	miR-21 overexpression	62.81	4.12	3.414E-05	0.636
	miR-155 overexpression	106.80	10.44	0.692	9.820E-03
	miR-21 & miR-155 overexpression	59.47	5.47	1.790E-04	
pMIR-RECK_miR-155-5p_BS2mut	miR-21 overexpression	53.02	2.49	2.115E-07	0.347
	miR-155 overexpression	99.35	4.35	0.896	1.912E-02
	miR-21 & miR-155 overexpression	64.18	9.50	8.670E-03	
pMIR-RECK_miR-155-5p_BS1&2mut	miR-21 overexpression	45.22	1.27	2.142E-09	0.095
	miR-155 overexpression	114.91	6.92	0.100	1.003E-03
	miR-21 & miR-155 overexpression	58.54	5.95	2.317E-04	
